# Mob at the Alms House

**Published:** 1847-10

**Authors:** 


*Mob at the Alms House.*—We have observed in the papers, some obscure allusions to a fracas at the New-York Alms House. The Annalist gives the following account of the affair under the caption which we have placed as the heading of this article:—

"An assistant physician, who had been discharged by the head of the Hospital, was invited, as we understand, by the remaining assistants, to take a seat with them at their table at dinner on Sunday. The remaining assistants, it seems, thought the discharge had been improperly issued, and intended this as an expression of their opinion. It was, of course, offensive to the other side, and the visitor was ordered away. He refused to go, when a subordinate officer was ordered to take him away. A combat ensued, in which some hard blows were passed before the expulsion was accomplished. Yesterday morning the assistant physician who invited the guest and resisted his expulsion, also received their discharge from Dr. Reese. *Jour. of Com.*, 18*th*. In a letter from Dr. Reese in the Tribune, dated Aug. 23d, 1847, he states that he had dismissed two of his assistants, and that two others had resigned. He denies that Dr. Reilay, 'whose intrusion at the public table, after repeated admonitions, at length rendered it necessary to remove him by force,' ever paid him a farthing. He received nothing with the other three who retired, having found them in the Hospital and retained them; and with respect to another, dismissed, the proportion of his advance payment due was returned. Dr. R. declares that he scrupulously refrained from making new appointments upon his taking charge of the Hospital, which would have entitled him to the usual fees, lest he might be accused of political proscription in reference to the appointments of his predecessor. The note closes with a complimentary card, exhibiting 'a spontaneous expression of opinion emanating from his faithful assistants,' voluntarily tendered to him to be used at his discretion. An investigation has been had of the matter before a Committee of the Common Council, marked, as we hear, by considerable acerbity on either side, but we have not heard that any report has been issued on the subject."

